# Maxillary Sinus Angiosarcoma in Cornelia de Lange Syndrome: A Case Report and Review of the Literature

**DOI:** 10.7759/cureus.82792

**Published:** 2025-04-22

**Authors:** Rishi Kondapaneni, Kaitlyn Florence, Laura Dooley, Filip Garrett

**Affiliations:** 1 Department of Otolaryngology, Head and Neck Surgery, University of Missouri School of Medicine, Columbia, USA; 2 Department of Pathology, University of Missouri School of Medicine, Columbia, USA

**Keywords:** angiosarcoma, chemotherapy, cornelia de lange syndrome, maxillary sinus angiosarcoma, neck dissection, nose and paranasal sinuses, radiation, sinus cancer

## Abstract

Angiosarcoma is a rare and aggressive subtype of soft-tissue sarcoma that typically originates from endothelial cells, often presenting in the head and neck (H&N) region. This case report aims to investigate a unique instance of sinonasal angiosarcoma in a patient with Cornelia de Lange syndrome (CdLS), a genetic disorder previously not associated with angiosarcoma, and to explore potential links between chronic rhinosinusitis (CRS) and sinonasal angiosarcoma.

A 22-year-old female patient with CdLS and a history of chronic sinusitis presented with epistaxis, facial pain, and a maxillary sinus mass. Imaging and biopsy suggested angiosarcoma, and surgical resection was performed. Our multidisciplinary tumor board recommended adjuvant chemotherapy. However, following consultation with an outside community hospital, a decision to proceed with observation was made. Persistent disease was identified on post-treatment imaging, leading to concurrent radiotherapy and weekly Taxol. There was no active disease upon follow-up.

To the best of our knowledge, this case represents the first report of angiosarcoma in a patient with CdLS. Although no direct link between CdLS and angiosarcoma has been established, CRS may create a microenvironment conducive to tumor development. Further research is necessary to better understand the relationship between CRS, genetic syndromes, and sinonasal angiosarcoma. Given the rarity and poor prognosis of sinonasal angiosarcoma, a multidisciplinary approach at academic centers is essential for optimal treatment.

## Introduction

Angiosarcoma is a rare subtype of soft-tissue sarcoma that originates from endothelial cells [1]. It accounts for 2% of soft-tissue sarcomas [2]. The most common sites are cutaneous lesions, particularly in the head and neck (H&N) region [2]. However, angiosarcoma may occur in any region, including the sinonasal or aerodigestive tract [2]. To date, there are only a handful of case reports documenting primary sinonasal angiosarcoma [1].

Angiosarcoma affects both men and women of any age with the common cutaneous presentation having predilection for men over 60 years of age [2]. Etiology is often sporadic with risk factors including chronic lymphedema, radiation history, environmental carcinogens, and various genetic syndromes such as neurofibromatosis, Ollier disease, Maffucci disease and Klippel-Trenaunay syndrome [2]. Its presentation is non-specific and depends upon the primary site of disease, with the most common symptoms for sinonasal angiosarcoma including nasal obstruction, malar edema and epistaxis [1-2].

Given the endothelial cell origin, angiosarcomas are often high-grade and aggressive with a poor prognosis [2]. Because it is rare, there is no standard treatment for sinonasal angiosarcoma [1]. Here, we present a case of angiosarcoma of the maxillary sinus in a patient with Cornelia de Lange syndrome (CdLS). To our knowledge, there are no reports, to date, of angiosarcoma with CdLS.

## Case presentation

**Figure 1:**
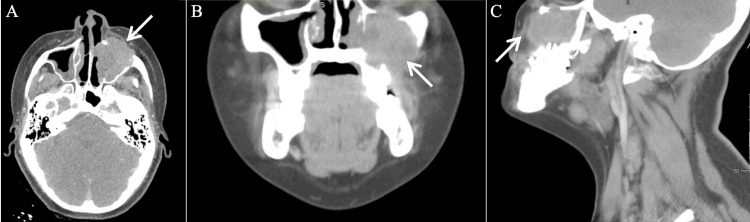
Initial axial (A), coronal (B), and sagittal (C) CT (neck) images demonstrating a left maxillary sinus mass with erosion of the anterior and posterior maxillary wall and left orbital floor with the rim intact

A 22-year-old female patient presented to our clinic with concerns for a left maxillary sinus mass. Her past medical history was notable for Cornelia de Lange syndrome and sinus surgery at 10 years of age due to recurrent acute sinusitis. She complained of epistaxis for the past one year. At the initial onset of epistaxis, she was evaluated by an outside otolaryngologist and was diagnosed with a benign nasal polyp. Approximately six months afterwards, she began having left facial and eye pain. She underwent CT of the neck that demonstrated a left sinus mass with erosion of the posterior maxillary wall and left orbital floor without lymphadenopathy (Figure 1). A biopsy revealed a malignant neoplasm most consistent with an angiosarcoma. She was then referred to our clinic. Upon clinical examination, she was found to have left midface edema with maxillary division of trigeminal nerve numbness.

**Figure 2:**
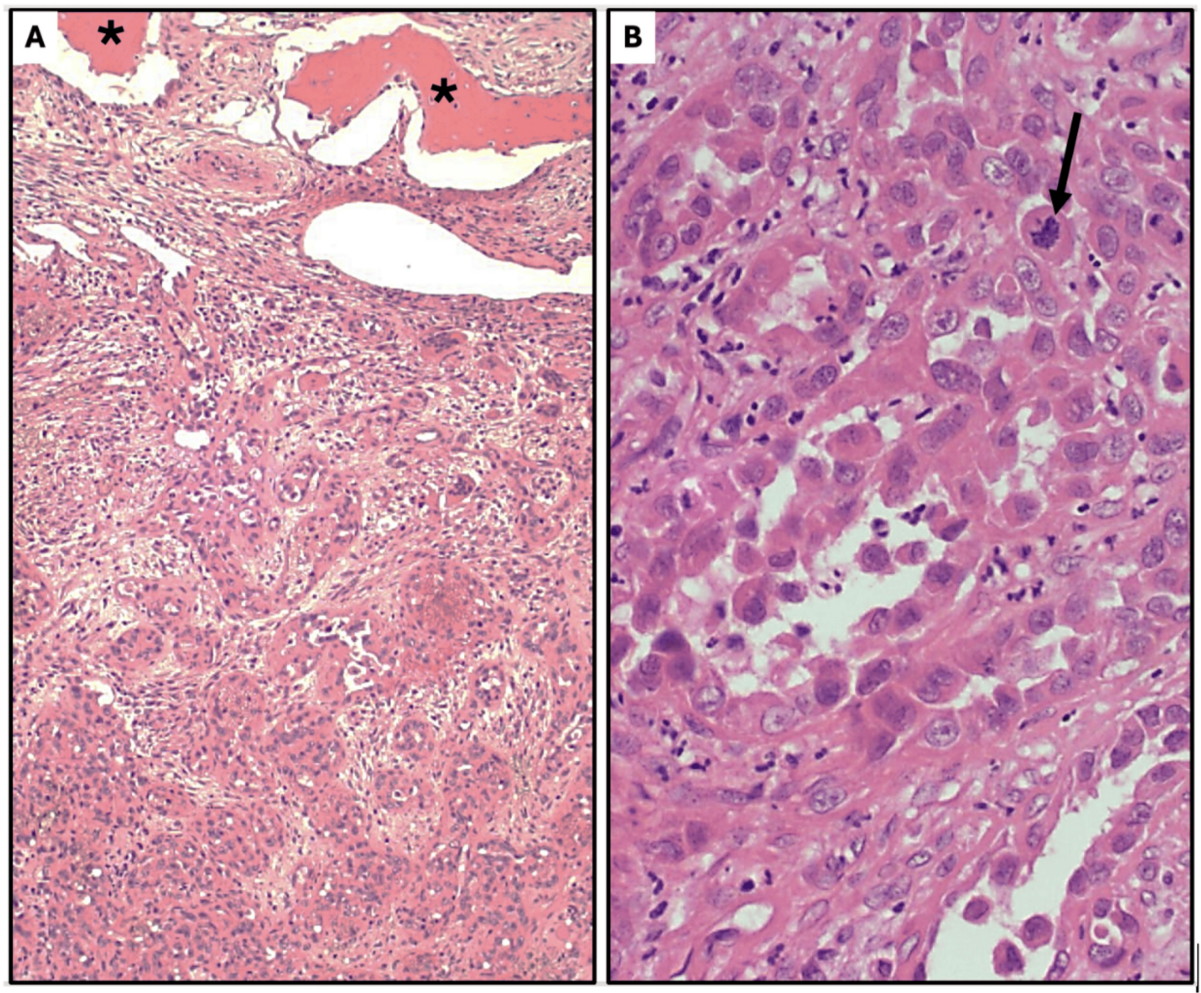
Sinus angiosarcoma, H&E. (A) Tumor invading the maxilla (*) through the orbital floor (pT4a); 100x. (B) Anastomosing vascular channels with markedly atypical tumor cells projecting into vascular spaces (“hobnail” pattern). Frequent atypical mitotic figures are present (arrow). Histologic grade, G3; 400x

A left total maxillectomy and left neck dissection were performed. Pathology demonstrated a high-grade angiosarcoma with negative margins and no targetable mutations on next-generation sequencing (Figure 2). All lymph nodes were negative for disease. Final staging was T4aN0M0 Stage IIIB. Our institution recommended adjuvant chemoradiation while a community institution recommended observation. The patient elected to proceed with observation.

**Figure 3:**
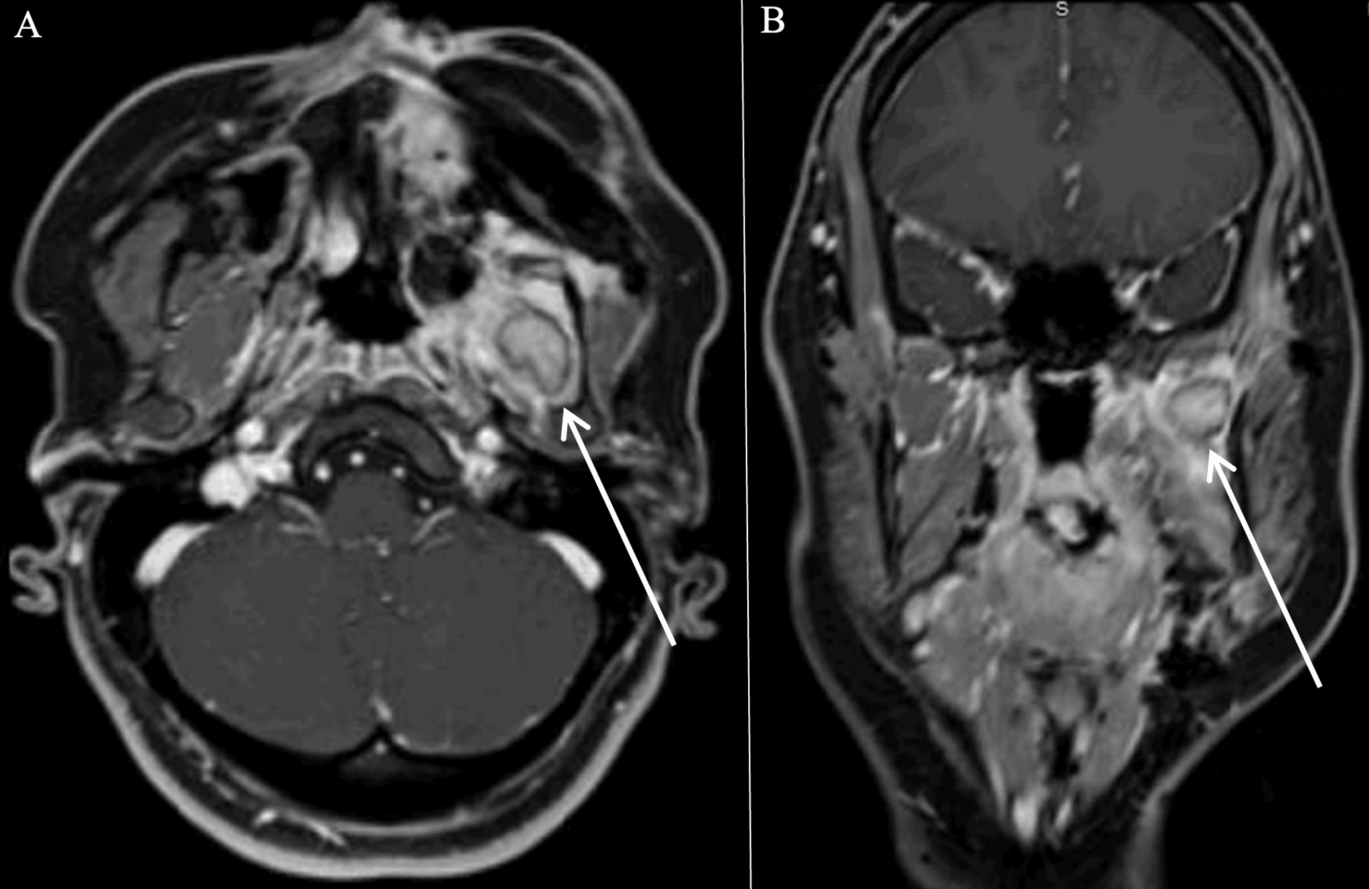
Three-month post-surgical resection imaging demonstrating a circumscribed mass within the left medial pterygoid muscle measuring 2 x 1.4 x 1.7 cm (AP x TRV x CC) with peripheral hypoenhancement on T1 axial (A) and coronal (B) MRI (face/neck/orbit) AP: anterior-posterior; TRV: transverse; CC: cranio-caudal

**Figure 4:**
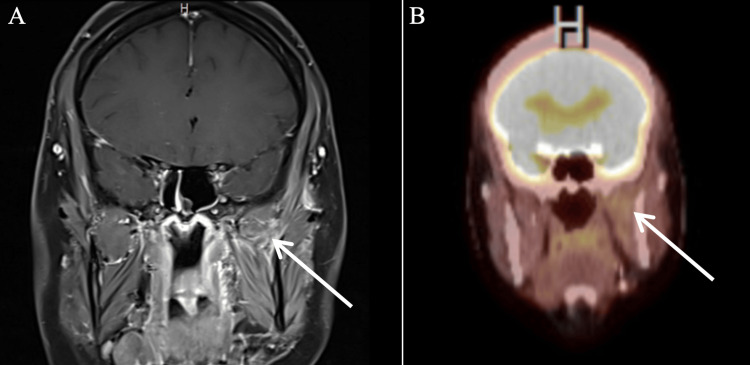
Three-month post-chemoradiation imaging demonstrating a persistent soft tissue density within the left masticator space on T1 coronal MRI of the face/neck/orbit (A). This area did not have FDG avidity upon concurrent PET/CT (B) FDG: fluorodeoxyglucose

On three-month post-treatment imaging, she was found to have an fluorodeoxyglucose (FDG)-avid lesion in the left masticator space on PET/CT and an ellipsoid, circumscribed mass in the left masticator space on MRI (Figure 3). She was diagnosed with persistent disease. She underwent seven cycles of paclitaxel with concurrent radiotherapy. Three-month post-chemoradiation treatment imaging was notable for persistent soft tissue density within the left masticator space, but it was without FDG avidity and deemed consistent with scarring (Figure 4).

## Discussion

Sinonasal angiosarcoma is a rare tumor with only 40 reported cases since 1976 (Table 1). None of these reports occurred in a patient with CdLS.

**Table 1:** Overview of reported cases of sinonasal angiosarcoma in the literature from 1976 to 2024 F: female; M: male; NR: not reported

Publication year	Author	Age	Sex	Location
1976	McClatchey et al. [3]	26	F	Maxillary sinus
1979	Bankaci et al. [4]	68	M	Maxillary sinus
1979	Sharma and Nawalkha [5]	10	M	Maxillary sinus
1986	Zachariades and Economopoulou [6]	68	F	Maxilla
1986	Zakrzewska [7]	58	M	Maxilla
1986	Panje et al. [8]	52	M	Nasal cavity, ethmoid
1988	Williamson and Ramsden [9]	48	M	Maxillary sinus
1989	Lanigan et al. [10]	73	M	Maxilla, maxillary sinus
1989	Kurien et al. [11]	38	M	Nasal cavity
1990	Solomons and Stearns [12]	33	M	Maxillary sinus
1990	Sobol et al. [13]	NR	NR	Maxillary sinus
1992	Kimura et al. [14]	8	M	Nasal cavity
2000	Velegrakis et al. [15]	72	M	Maxillary sinus
2001	Wong et al. [16]	34	F	Sphenoid sinus
2002	Triantafillidou et al. [17]	50	F	Maxillary sinus
2004	Yamaguchi et al. [18]	53	M	Maxilla
2005	Oliveira et al. [19]	56	M	Nasal cavity
2006	Fukushima et al. [20]	55	M	Nasal cavity
2007	Nelson and Thompson [21]	NR	NR	Nasal cavity
2007	Nelson and Thompson [21]	NR	NR	Nasal cavity
2007	Nelson and Thompson [21]	NR	NR	Nasal cavity
2007	Nelson and Thompson [21]	NR	NR	Nasal cavity
2007	Nelson and Thompson [21]	NR	NR	Nasal cavity
2007	Nelson and Thompson [21]	NR	NR	Nasal cavity
2007	Nelson and Thompson [21]	NR	NR	Nasal cavity
2007	Nelson and Thompson [21]	NR	NR	Nasal cavity
2007	Nelson and Thompson [21]	NR	NR	Maxillary sinus
2007	Nelson and Thompson [21]	NR	NR	Maxillary sinus
2009	Treviño-González et al. [1]	33	M	Nasal cavity
2012	Deenadayal et al. [22]	29	M	Maxillary sinus
2013	Gravvanis et al. [23]	68	M	Nasal cavity
2014	Tomovic et al. [24]	21	F	Frontal sinus
2015	Es-Sbissi et al. [25]	53	M	Nasal cavity
2015	Deshmukh et al. [26]	50	F	Nasal cavity
2015	Sun et al. [27]	29	F	Maxillary sinus
2015	Mullins and Hackman [28]	52	M	Maxillary sinus
2018	Chung et al. [29]	30	F	Maxillary sinus
2023	Kou and Cheng [30]	70	F	Maxillary sinus
2023	Chai et al. [31]	40	M	Maxillary sinus
2024	Kimura et al. [32]	74	F	Nasal cavity

CdLS is a rare genetic condition due to mutations in cohesin structural and regulatory genes [33]. It often occurs due to sporadic gene mutations; however, it can also be inherited as an autosomal dominant or X-linked dominant condition. Patients with CdLS have a wide range of phenotypes. The classic phenotype includes distinctive facial features, intellectual disability, growth delay and upper limb reduction [34]. Diagnosis is based upon physical examination and genetic testing [33].

Otolaryngologic disorders most seen in CdLS include eustachian tube dysfunction, chronic middle ear effusion, mixed hearing loss, speech delay, cleft palate, dysphagia, gastroesophageal reflux and obstructive sleep apnea [34]. Patients with CdLS have also been found to have an increased risk of chronic rhinosinusitis (CRS) with some reports noting development of childhood nasal polyps [34]. In severe cases, such as the patient in this case, pediatric sinus surgery is indicated. However, there is limited literature regarding the prevalence and prognosis of CRS. Despite CdLS occurring secondary to genetic defects of cohesin and regulatory genes, a recent retrospective study found no association for malignancy in individuals with CdLS; however, further studies in larger populations are needed [33].

The etiology for angiosarcoma is often sporadic with known risk factors such as chronic lymphedema, radiation history, environmental carcinogens and various genetic syndromes such as neurofibromatosis and Von Hippel-Lindau, but not CdLS [2]. The correlation between chronic lymphedema and angiosarcoma was initially seen in patients with a history of breast cancer who developed lymphedema after mastectomy and subsequent angiosarcoma; this phenomenon is called Stewart-Treves syndrome [2].

Although there are no reports of CRS contributing to angiosarcoma, CRS has been associated with insufficient clearing of the nasal mucosa through lymphatics resulting in fluid retention and possible polyp formation [35]. This creates an environment similar to chronic lymphedema. Upon reviewing the reported sinonasal angiosarcoma cases, a history of CRS was noted in three cases, with the other cases not commenting upon a history of CRS. Thus, it is possible that there is a correlation between the chronic inflammatory state of rhinosinusitis and development of sinonasal angiosarcoma. However, further research is needed.

There are no comprehensive guidelines specifically for sinonasal or H&N angiosarcoma. Currently, all forms of angiosarcoma are treated in a similar manner. For Stage III angiosarcoma seen in this case, the National Comprehensive Cancer Network recommends surgical resection followed by radiation and possible systemic therapy. However, the guidelines are based on low evidence and do not distinguish between various sarcoma subtypes. Therefore, given the paucity of formal guidelines, a multidisciplinary approach at an academic center is vital as there remains a variety in treatment approaches.

Despite the rarity and undifferentiated guidelines, it has been reported that sinonasal angiosarcoma has a better survival rate of 22% compared to cutaneous H&N angiosarcoma with a survival rate of 12% at five years [1]. This further contributes to the notion that choosing treatment strategies based on the subtype may be beneficial.

## Conclusions

Angiosarcomas are rare with a poor prognosis. There may be an association between angiosarcoma and CRS; however, larger studies are needed in relation to sinonasal angiosarcoma as well as CdLS to assess risk factors. Given the rarity and spectrum of the disease, improved guidelines for the treatment of H&N angiosarcoma are needed. Due to the lack of guidelines, it is important to manage rare tumors, especially in the setting of syndromic conditions, using a multidisciplinary approach.
